# Ultrasound-Activated BiOI/Ti_3_C_2_ Heterojunctions in 3D-Printed Piezocatalytic Antibacterial Scaffolds for Infected Bone Defects

**DOI:** 10.3390/ma18153533

**Published:** 2025-07-28

**Authors:** Juntao Xie, Zihao Zhang, Zhiheng Yu, Bingxin Sun, Yingxin Yang, Guoyong Wang, Cijun Shuai

**Affiliations:** 1 Institute of Bioadditive Manufacturing, Jiangxi University of Science and Technology, Nanchang 330013, China; xjt19871948043@163.com (J.X.); 6720231542@mail.jxust.edu.cn (Z.Z.); sunbingxin@jxust.edu.cn (B.S.); 9519930003@jxust.edu.cn (Y.Y.); 2 College of Mechanical and Electrical Engineering, Jiaxing Nanhu University, Jiaxing 314001, China; yuzhiheng@jxnhu.edu.cn; 3 State Key Laboratory of Precision Manufacturing for Extreme Service Performance, College of Mechanical and Electrical Engineering, Central South University, Changsha 410083, China

**Keywords:** bacterial infection, BiOI/Ti_3_C_2_, piezocatalytic therapy, scaffold

## Abstract

Piezocatalytic therapy (PCT) is a promising strategy for combating implant-associated infections due to its high tissue penetration depth and non-invasive nature. However, its catalytic efficiency remains limited by inefficient electron–hole separation. In this work, an ultrasound-responsive heterojunction (BiOI/Ti_3_C_2_) was fabricated through in situ growth of bismuth iodide oxide on titanium carbide nanosheets. Subsequently, we integrated BiOI/Ti_3_C_2_ into poly(e-caprolactone) (PCL) scaffolds using selective laser sintering. The synergistic effect between BiOI and Ti_3_C_2_ significantly facilitated the redistribution of piezo-induced charges under ultrasound irradiation, effectively suppressing electron–hole recombination. Furthermore, abundant oxygen vacancies in BiOI/Ti_3_C_2_ provide more active sites for piezocatalytic reactions. Therefore, it enables ultrahigh reactive oxygen species (ROS) yields under ultrasound irradiation, achieving eradication rates of 98.87% for *Escherichia coli* (*E. coli*) and 98.51% for *Staphylococcus aureus* (*S. aureus*) within 10 minutes while maintaining cytocompatibility for potential tissue integration. This study provides a novel strategy for the utilization of ultrasound-responsive heterojunctions in efficient PCT therapy and bone regeneration.

## 1. Introduction

Deep tissue implant-associated infections pose a significant clinical challenge. Their persistent biofilm formation and escalating antibiotic resistance frequently lead to implant failure and costly revision surgeries [1]. Traditional antibiotic treatments face limitations due to poor biofilm penetration and rapid microbial resistance development, severely restricting their effectiveness [2,3]. Consequently, alternative approaches that overcome antibiotic limitations are attracting growing interest. Among these, reactive oxygen species (ROS)-based non-antibiotic strategies hold promise. However, their clinical application is hindered by difficulties in achieving precise spatiotemporal control over targeted ROS generation in deep tissues [4,5]. Recently, piezocatalytic therapy (PCT) activated by non-invasive low-intensity ultrasound (LIU) has emerged. This groundbreaking modality can overcome these barriers [6,7]. PCT enables deep tissue penetration and localized ROS production through mechanical energy-driven catalytic reactions with minimal off-target toxicity [8]. This approach offers unique advantages for eradicating deep-seated infections, as it harnesses mechanical stimuli to trigger catalytic processes, enabling sustained and controlled ROS generation.

In recent years, studies of ultrasound-responsive piezoelectric materials in PCT research have emerged rapidly. Among various piezoelectric materials, bismuth oxyiodide (BiOI) is a promising piezocatalyst owing to its exceptional piezoelectric coefficient (d_33_ ≈ 25 pm/V) and visible-light absorption capacity [9]. These properties facilitate ultrasound-triggered ROS generation. Its narrow bandgap (~1.38 eV) also promotes additional charge carrier generation via sonoluminescence [10]. However, BiOI’s practical application is limited by rapid electron–hole recombination and inefficient charge separation kinetics, leading to low ROS yields under LIU—typically below 0.5 μM/min. For example, Wang et al. [11] reported only 45% bacterial inhibition using BiOI nanosheets under 1.5 W/cm^2^ ultrasound, attributed to severe charge recombination. Similarly, achieving 80% *Staphylococcus aureus* eradication with unmodified BiOI required extended treatment (30 min), highlighting the critical need to enhance charge separation and prolong carrier lifetimes [12]. Addressing this fundamental limitation requires innovative heterojunction engineering to optimize interfacial charge dynamics and enhance ROS production efficiency.

Recent advances in defect modulation and heterojunction design demonstrate potential for mitigating recombination losses. For instance, Ti_3_C_2_ MXene, a two-dimensional transition metal carbide with ultrahigh electrical conductivity and abundant surface adsorption sites, has been employed as an effective electron acceptor [13]. Synergistic platforms such as Ti_3_C_2_@TiO_2_ have demonstrated significant enhancements in charge separation, achieving 95% *E. coli* eradication within 5 minutes under ultrasound [14]. Moreover, oxygen-vacancy-engineered ZnO/Ti_3_C_2_ heterojunctions reported by Chen et al. reduced recombination by 70% and doubled ROS yields compared to pure ZnO [15]. Building on these insights, we hypothesized that integrating BiOI with Ti_3_C_2_ would create a built-in electric field to direct piezoelectric electrons unidirectionally from BiOI to Ti_3_C_2_, suppressing recombination. Additionally, engineered oxygen vacancies (OVs) at the heterointerface act as efficient electron traps, prolonging carrier lifetimes by over threefold [16,17]. We anticipated that this combined approach—Schottky junction formation coupled with defect-mediated electron trapping—would synergistically enhance ROS generation under LIU stimulation.

**Figure 1:**
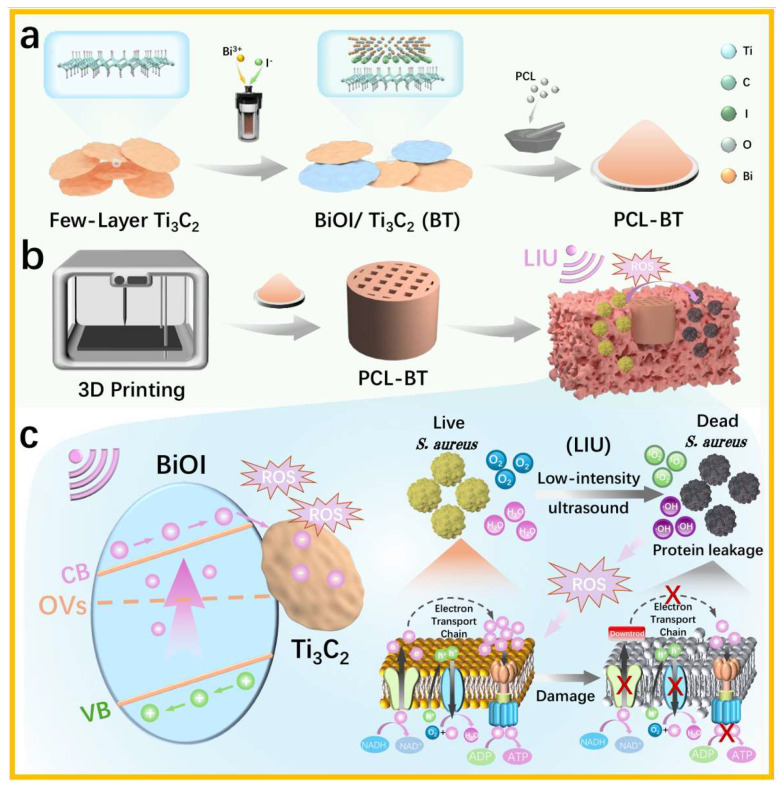
(**a**) The synthesis process of PCL-BT. (**b**) A schematic diagram of the preparation of the PCL-BT scaffold. (**c**) AN electrochemical mechanism diagram of BiOI/Ti_3_C_2_ under low-intensity ultrasound (LIU) and the corresponding antibacterial mechanism (*S. aureus* as an example).

Specifically, within the microenvironment of bone defects during repair, bacteria rapidly multiply and form biofilms, posing a major threat to the postoperative management of bone implants [18]. Therefore, we aimed to utilize 3D printing for fabricating a bone scaffold with ultrasound-responsive antibacterial properties. In this work, we engineered an oxygen-vacancy-rich BiOI/Ti_3_C_2_ heterojunction via in situ solvothermal synthesis (Figure 1a). The heterojunction was subsequently integrated into spatially programmable three-dimensional polycaprolactone (PCL) scaffolds through selective laser sintering (Figure 1b). Comprehensive physicochemical characterization (morphology, crystal structure, defect density) and electrochemical assessments (charge transfer kinetics, interfacial resistance) were performed to elucidate the structural and electrochemical properties of BT. We further evaluated ROS generation capability and antibacterial efficacy against Gram-negative and Gram-positive bacteria under LIU stimulation (Figure 1c), alongside conducting cytocompatibility tests to assess tissue regeneration potential. This multifaceted investigation aims to establish a highly efficient, controllable piezocatalytic platform for bone defect infections.

## 2. Materials and Methods

### 2.1. Materials

The powdered polycaprolactone (PCL) (Mw = 100 kDa) (Jusuke Biomaterials Co., Ltd, Shenzhen, China). MXene (single-layer Ti_3_C_2_ powder), bismuth nitrate pentahydrate (Bi(NO_3_)_3_·5H_2_O), and potassium iodide (KI) were purchased from the same company (Aladdin Biochemical Technology Co., Ltd, Shanghai, China). Hydrochloric acid (HCl) and acetic acid (CH_3_COOH) were purchased from the same company (Maclean Biochemical Technology Co., Ltd, Shanghai, China).

### 2.2. Preparation of BiOI/Ti_3_C_2_

BiOI/Ti_3_C_2_ (BT) was fabricated via a solvothermal method. In brief, 2.5 mmol Bi(NO_3_)_3_·5H_2_O was dissolved in 10 mL deionized water, followed by the addition of 50 mg monolayered Ti_3_C_2_. The mixture was ultrasonicated for 30 minutes and stirred for 3 h to form suspension A. Separately, solution B was prepared by dissolving 2.5 mmol KI in 20 mL deionized water. Following dropwise incorporation of solution B into suspension A during 1 h of continuous agitation, the resultant mixture was subjected to hydrothermal synthesis in a 100 mL Teflon-lined autoclave maintained at 150 °C for 6 h in an oven. After cooling to room temperature, the product was isolated by centrifugation (12,000 rpm, 10 min/cycle), extensively rinsed with deionized water, lyophilized overnight, and ground into BT powder.

### 2.3. Physicochemical Characterizations of BT

Sample morphology and microstructure were analyzed using a JEM-F200 transmission electron microscope (TEM, JEOL Ltd., Tokyo, Japan) equipped with energy-dispersive X-ray spectroscopy (EDS). Crystal structure and phase composition were assessed via XRD (Bruker, Karlsruhe, Germany), while zeta potentials were measured using a Nano Zetasizer ZEN3600 (Instruments Ltd., Malvern, UK). Reactive oxygen species (ROS) were detected and characterized using electron paramagnetic resonance (EPR) spectroscopy (EMX Plus, Bruker, Karlsruhe, Germany). Surface composition and chemical states were characterized by X-ray photoelectron spectroscopy (AXIS Ultra, Thermo Scientific, Waltham, MA, USA). UV-Vis-NIR absorption spectra (200–1000 nm) were acquired with a Varioskan LUX microplate reader (Thermo Fisher Scientific, Waltham, MA, USA). Transient fluorescence analysis was conducted with a steady-state/transient fluorescence spectrometer (FluoroLog-3, Horiba Scientific, Kyoto, Japan).

### 2.4. Electrochemical Measurement

We employed a three-electrode configuration to evaluate the electrochemical properties of the samples. A 50 mL aqueous electrolyte was prepared by dissolving 0.8231 g potassium ferricyanide (K_3_[Fe(CN)_6_]) and 0.372 g potassium chloride (KCl) in deionized water under vigorous stirring. An Ag/AgCl reference electrode and platinum wire counter electrode were utilized. The working electrode was fabricated by ultrasonically dispersing 10 mg sample in 1 μL ethanol to form a homogeneous suspension. The suspension was drop-cast onto ITO-coated glass and dried at 60 °C. Electrochemical impedance spectroscopy (EIS) measurements for both BiOI and BT were conducted using a CHI660E workstation (CH Instruments, Austin, TX, USA). The response of piezoelectric current varying with time was recorded under low-intensity ultrasound (LIU, 1.0 W/cm^2^, 1 MHz, 50% duty cycle).

### 2.5. Preparation of Composite Scaffolds

We fabricated porous scaffolds using selective laser sintering (SLS) [19]. Bismuth oxyiodide (BiOI) and BT composite powders were synthesized through a sequential process, involving dissolution, magnetic stirring, ultrasonication, centrifugation, and freeze-drying. Preliminary experiments confirmed that a BT content exceeding 5 wt.% compromised scaffold integrity; thus, the optimal BT loading was fixed at 4 wt.% in the composite powder. The SLS parameters were optimized as follows: laser power, 6 W; scan spacing, 0.10 mm; pulse width, 10 μs. During printing, the platform temperature was maintained at 120 °C, and the laser beam scanned the powder bed at a speed of 120 mm/s. Cylindrical scaffolds with interconnected porous architectures were successfully obtained and labeled as PCL, PCL-BiOI, and PCL-BT, corresponding to pure poly(e-caprolactone) (PCL), BiOI-loaded, and BT-loaded composites, respectively.

### 2.6. Measurement of Extracellular ROS

Hydroxyl radical (•OH) generation by the scaffolds was assessed using methylene blue (MB) as a probe. Briefly, PCL, PCL-BiOI, and PCL-BT scaffolds were submerged in MB solution and exposed to low-intensity ultrasound (UT1021, Dongdixin Technology Co., Shenzhen, China) for 0–10 minutes (intervals: 2 min). Absorbance spectra (500–800 nm) were documented utilizing a UV-1800 spectrophotometer (UROP Scientific Instruments, Shanghai, China).

Singlet oxygen (^1^O_2_) production was assessed using 1,3-diphenylisobenzofuran (DPBF) as the indicator probe. Using equivalent procedures to MB preparation, DPBF solution was formulated. Samples were immersed in DPBF solution and sonicated intermittently (0–10 min, 2 min intervals). Post-sonication, absorbance measurements (350–500 nm) were performed using quartz cuvettes.

### 2.7. In Vitro Bacterial Inhibition Testing

The in vitro antibacterial efficacy of the scaffolds was evaluated against the Gram-negative bacterium *Escherichia coli* (ATCC 25922) and the Gram-positive bacterium *Staphylococcus aureus* (ATCC 25923). The liquid culture medium formulation comprised 1% peptone, 1% NaCl, and 0.5% yeast extract, with deionized water constituting the remainder (97.5%). For solid medium, 1.4% (*w*/*v*) agar was incorporated. Sterilization was achieved by autoclaving at 120 °C for 20 minutes. Bacterial suspensions (1 μL) were inoculated into 1 mL liquid medium to prepare bacterial cultures. After UV sterilization for 30 min, scaffolds were immersed in the bacterial suspensions and incubated at 37 °C for 6 h. The samples (scaffolds with adherent bacteria) were then divided into two groups: one exposed to LIU (1.0 W/cm^2^, 10 min) and the other without ultrasound (control group).

After treatment, 10^4^-diluted bacterial suspensions were spread-plated using 25 μL aliquots on culture agar. Following 16 h incubation at 37 °C, colony clusters were documented photographically and quantified via colony-forming unit (CFU) enumeration. Antibacterial rates were calculated by Equation (1) [20]:

Antibacterial rate (%) = [(CFU_control_–CFU_experimental_)/CFU_control_] × 100% (1)

where CFU_control_ and CFU_experimental_ represent CFU counts for untreated and ultrasound-treated groups, respectively. All experiments were performed in triplicate to ensure statistical reliability.

### 2.8. Evaluation of Bacterial Activity in Vitro

#### 2.8.1. ROS Staining

PCL, PCL-BiOI, or PCL-BT scaffolds were individually immersed in the bacterial suspension and incubated for 6 hours in individual centrifuge tubes. The bacterial suspensions were centrifuged at 4 °C (Eppendorf Centrifuge 5424 R, Eppendorf, Hamburg, Germany) and resuspended in a staining solution containing DCFH-DA (2’,7’-dichlorodihydrofluorescein diacetate) diluted in DMEM (Dulbecco’s Modified Eagle Medium) at a 1:1000 ratio. The compound was incubated under dim conditions for 30 minutes and centrifuged to remove excess dye. Bacterial pellets underwent triple-washing with phosphate-buffered saline (PBS) for residual stain removal, followed by final resuspension in fresh PBS. A 10 μL aliquot of the stained suspension was mounted on a glass slide and imaged using a fluorescence microscope (Nikon Eclipse Ti2, Tokyo, Japan) with appropriate excitation/emission filters (Ex/Em = 488/525 nm).

#### 2.8.2. LIVE/DEAD Staining

PCL, PCL-BiOI, or PCL-BT scaffolds were individually immersed in the bacterial suspension and incubated for 6 hours in individual centrifuge tubes. The suspensions were centrifuged at 4 °C (Eppendorf Centrifuge 5424 R, Eppendorf, Hamburg, Germany) and washed twice with phosphate-buffered saline (PBS). A live/dead staining solution containing DAPI (4’,6-diamidino-2-phenylindole), assay buffer, and PI (propidium iodide, Biohao Biotechnology Co., Ltd., Shanghai, China) was incorporated into the pelleted bacteria. Incubation proceeded for 30 minutes under dark conditions and centrifuged to remove excess dye. Bacteria were washed twice with PBS to eliminate residual staining agents. Resuspended bacteria (10 μL) were deposited onto glass slides for fluorescence microscopic visualization using an Olympus BX51 system (Olympus Corporation, Tokyo, Japan) with DAPI (358/461 nm Ex/Em) and PI (535/617 nm Ex/Em) filter sets.

### 2.9. Cell Cytotoxicity

To assess cytotoxicity, L929 mouse fibroblasts were seeded onto PCL, PCL-BiOI, or PCL-BT scaffolds and incubated. The scaffolds were placed in each well of a 96-well plate, and L929 cells were inoculated on the scaffolds. After 1 day and 3 days of culture, at the predetermined time points, Calcein-AM (Beituo, Shanghai, China) and PI (propidium iodide, Baohao Biotechnology Co., Ltd., Shanghai, China) were used for live/dead fluorescence staining of the L929 cells that had been incubated with the scaffolds, and the staining results were observed using a fluorescence microscope (Nikon Eclipse Ti2, Tokyo, Japan).

### 2.10. Statistical Analysis

For each group, the sample size was established as n = 3. The results are presented as the mean ± standard deviation (SD). Two-group comparisons employed Student’s *t*-test; multi-group analyses used one-way ANOVA. * represents *p* < 0.05, while *** represents *p* < 0.001. The results of * *p* < 0.05 and *** *p* < 0.001 was considered to be statistically significant. All analyses were performed in Image J and Origin Pro 2021 (Origin Lab, Northampton, MA, USA).

## 3. Results and Discussion

### 3.1. Fabrication and Characterization of BT

For the synthesis of BT, initially, few-layer Ti_3_C_2_ nanosheets were uniformly dispersed via ultrasonication. Bi^3+^ and I^−^ precursors were subsequently introduced to form a homogeneous solution. The negatively charged Ti_3_C_2_ surface facilitated electrostatic adsorption of Bi^3+^, enabling bottom–up in situ growth of BiOI nanosheets. Owing to the two-dimensional (2D) morphology and high surface reactivity, Ti_3_C_2_ provided abundant nucleation sites for BiOI, resulting in a tightly integrated BT heterostructure.

**Figure 2:**
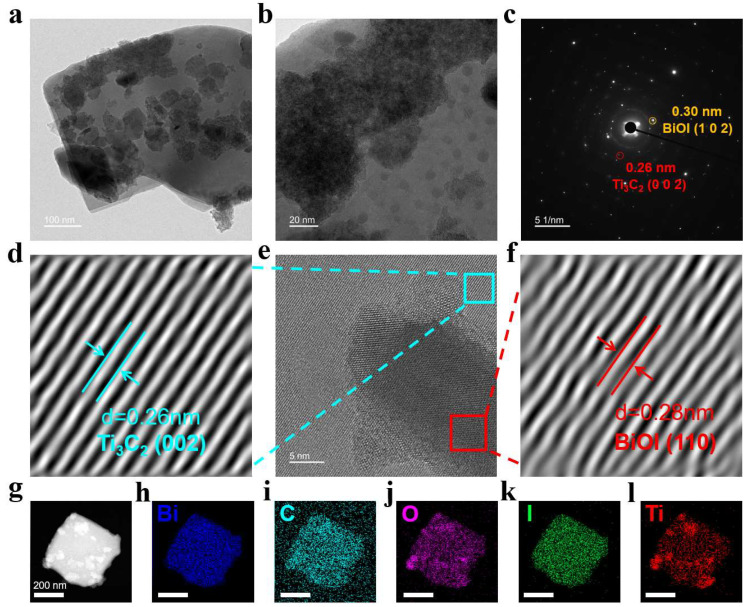
BT’s characterization: (**a**) TEM image, and (**b**) local magnification of TEM image. (**c**) SAED patterns of BiOI/Ti_3_C_2_. (**d**) Fourier transform images of Ti_3_C_2_. (**e**) HRTEM, and (**f**) corresponding Fourier transform images of BiOI. (**g**–**l**) EDS analysis of BiOI/Ti_3_C_2_.

TEM images (Figure 2a,b) revealed mutually stacked two-dimensional layered structures of varying sizes, consistent with the formation of the BT heterostructure. Figure 2c displays the selected area electron diffraction (SAED) rings, assignable to the (002) plane of Ti_3_C_2_ and the (102) plane of BiOI. High-resolution transmission electron microscopy (HRTEM) analysis confirmed the crystal structure (Figure 2e). Inverse Fourier transform (IFFT) measurements revealed lattice spacings of 0.26 nm and 0.28 nm, corresponding to the (002) plane of Ti_3_C_2_ (Figure 2d) and the (110) plane of BiOI (Figure 2f), respectively. EDS analysis further confirmed the uniform distribution of Bi, C, O, I, and Ti within the BiOI/Ti_3_C_2_ composite (Figure 2g–l). These results confirm the successful synthesis of BT.

**Figure 3:**
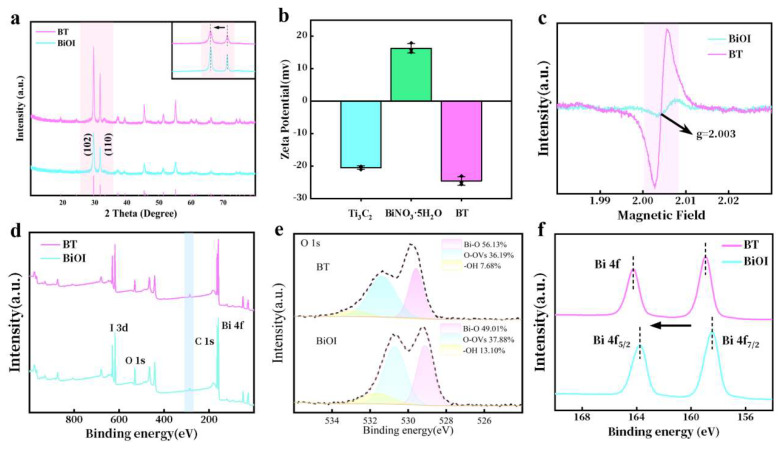
(**a**) XRD patterns of BiOI and BT. (**b**) Measurement of zeta potential. (**c**) Low-temperature ESR of BiOI and BT. (**d**) Full spectrum of XPS for different samples. (**e**) Fitted spectrum of O 1s and (**f**) Bi 4f of BiOI and BT.

X-ray diffraction (XRD) analysis (Figure 3a) confirmed the tetragonal crystal phase of BiOI (PDF 10–0445) [21], while the absence of distinct Ti_3_C_2_ peaks in BT indicated its homogeneous dispersion within the heterostructure. The subtle shift of the BiOI diffraction peaks towards lower angles suggests an augmentation in the interlayer spacing. This phenomenon is attributed to the interface strain that arises from the incorporation of Ti_3_C_2_. Determination of the zeta potential (Figure 3b) indicates that when Ti_3_C_2_ with strong negative charge is ultrasonically dispersed in an aqueous solution and fully mixed with Bi(NO_3_)_3_·5H_2_O, the positively charged Bi(NO_3_)_3_·5H_2_O can self-assemble through electrostatic forces and deposit on Ti_3_C_2_, serving as the precursor for the synthesis of BiOI. Electron spin resonance (ESR) analysis (Figure 3c) further confirmed oxygen vacancy formation, with BT exhibiting a significantly stronger signal at g = 2.003 compared to pristine BiOI, indicating an enhanced defect density.

X-ray photoelectron spectroscopy (XPS) survey spectra (Figure 3d) exhibited characteristic peaks corresponding to Bi 4f, I 3d, O 1s, and C 1s. There is no distinguishable Ti 2p peak in the BT group, which can be attributed to the decrease in the concentration of Ti 2p or its uniform dispersion in BT. Deconvolution of the high-resolution O 1s spectrum identified three distinct peaks, as presented in Figure 3e. These peaks were assigned to lattice oxygen (Bi-O at 529.8 eV), adsorbed oxygen originating from surface oxygen vacancies (OVs-O at 531.0 eV), and chemisorbed oxygen species (BiOH at 532.8 eV) [22]. Notably, the semi-quantitative analysis of the peak fitting revealed an increase in the Bi-O content alongside a reduction in the concentration of oxygen vacancies (OVs), thereby corroborating the critical role of oxygen vacancies in charge compensation. High-resolution Bi 4f spectra (Figure 3f) displayed binding energy shifts, indicative of interfacial charge redistribution between BiOI and Ti_3_C_2_. Collectively, these characterizations validate the successful synthesis of a defect-rich heterojunction with optimized interfacial charge transfer properties.

### 3.2. Characterization of Piezoelectric Properties of BT

**Figure 4:**
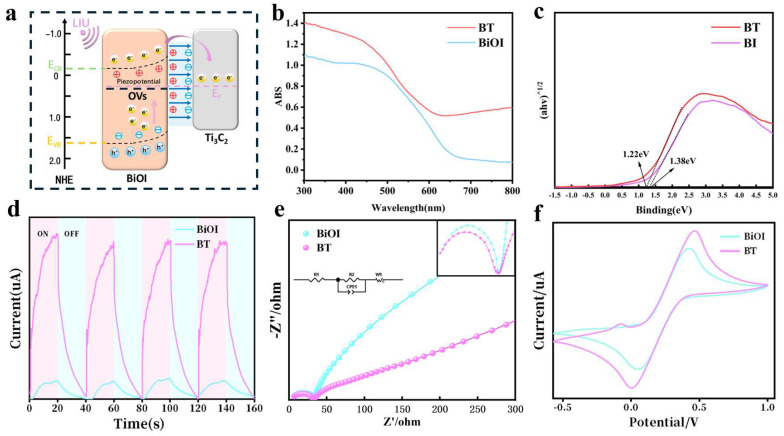
Characterization of electrochemical properties: (**a**) piezoelectric catalytic mechanism diagram of BT, (**b**) UV–Vis DRS, and (**c**) tauc curves of BiOI and BT. (**d**) Piezoelectric current and (**e**) electrical impedance curves of BiOI and BT. (**f**) CV curve graphs of BiOI and BT.

The enhanced piezocatalytic performance of the BiOI/Ti_3_C_2_ heterojunction (BT) under LIU arises from optimized interfacial charge dynamics and defect engineering (Figure 4a). Mechanical stress induced by LIU triggers piezoelectric polarization in the non-centrosymmetric BiOI lattice, generating localized potentials that spatially separate electrons (e^−^) and holes (h^+^) [23,24]. Simultaneously, ultrasound-induced mechanical stress promotes rapid electron excitation from the valence band (VB) to the conduction band (CB). Concurrently, the Schottky junction at the BiOI/Ti_3_C_2_ interface directs e^−^ transfer to the conductive Ti_3_C_2_ matrix, effectively suppressing recombination. UV-Vis diffuse reflectance spectroscopy (DRS) indicates that in the presence of surface oxygen vacancies, the absorption edge of BT has shifted towards the red compared to the original BiOI (Figure 4b), corresponding to a reduction in the bandgap energy (1.22 eV for BiOI and 1.38 eV for BiOI, as shown in Figure 4c). This expansion of light absorption into the near-infrared region, combined with the interface polarization effect, promotes the efficient generation of carriers under low-intensity ultrasound [25,26]. The results of the piezoelectric current measurement show that the current density of BT is 2.8 times higher (Figure 4d), confirming enhanced charge separation.

Under low-intensity ultrasound, the electrochemical impedance spectroscopy (EIS, Figure 4e) shows that the semicircular arc of BT is smaller, indicating that BiOI/Ti_3_C_2_ generates more electrons under ultrasonic action. This leads to more efficient carrier migration, resulting in significantly faster separation of electron–hole pairs. These findings further confirm that BT exhibits improved sonodynamic performance after the in situ formation of BiOI. This might be due to the carbon matrix remaining on Ti_3_C_2_, which shows enhanced conductivity. Consequently, this enhances electron–hole pair separation efficiency during sonodynamic processes, thus improving the composite’s sonodynamic performance. Furthermore, cyclic voltammetry (CV, Figure 4f) exhibits a distinct reduction peak at 0.02 V (vs. Ag/AgCl) for BT, in contrast to the broad peak observed at 0.12 V for BiOI. This signifies accelerated oxygen reduction kinetics (O_2_ → •O_2_^−^) and effective •OH utilization (oxidation peak at +0.45 V). Collectively, BT’s hierarchical design—integrating oxygen vacancies, Schottky junctions, and dual-field effects—optimizes ROS generation (•OH, ^1^O_2_) and charge transfer efficiency, establishing a paradigm for non-antibiotic antimicrobial therapies in deep tissue applications.

### 3.3. Sonodynamic Performance of the Scaffolds

**Figure 5:**
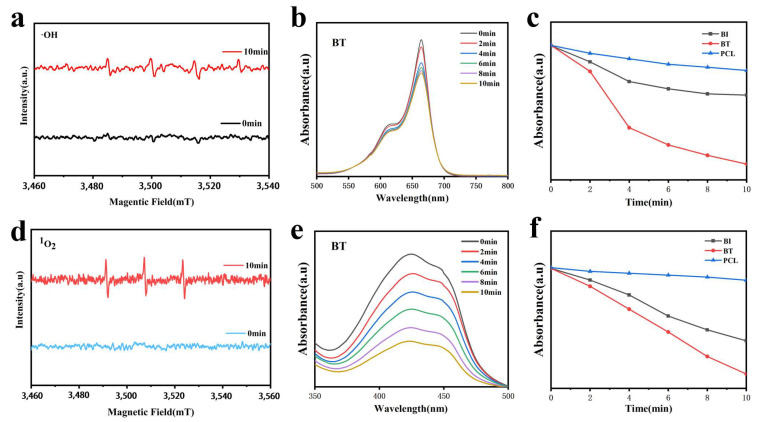
(**a**) MB absorbance of PCL-BT scaffold under low-intensity ultrasound. (**b**) Comparison chart of MB absorbance values of PCL, PCL-BiOI, and PCL-BT scaffolds. (**c**) ESR spectra of ·OH. (**d**) DPBF absorbance of PCL-BT scaffold under low-intensity ultrasound treatment. (**e**) Comparison chart of DPBF absorbance values of PCL, PCL-BiOI, and PCL-BT scaffolds. (**f**) ESR spectra of ^1^O2.

Electron spin resonance (ESR) experiments verified the generation of hydroxyl radicals (•OH) under LIU (1.0 W/cm^2^, 10 min), as shown in Figure 5a. A distinct •OH ESR signal, exhibiting the diagnostic 1:2:2:1 quartet upon DMPO trapping, was observed in the PCL-BT scaffold, confirming hydroxyl radical generation [27]. We further evaluated the sonodynamic activity of the scaffolds by quantifying hydroxyl radical (•OH) generation under LIU, using methylene blue (MB) as a probe [28]. MB absorption at 664 nm decreased proportionally due to •OH-mediated oxidative degradation. As shown in Figure 5b, the PCL-BT scaffold exhibited a significant reduction in MB absorbance following LIU treatment (0–10 min, with 2 min intervals), confirming its efficient •OH production capability. In contrast, pure PCL scaffolds showed negligible changes, while PCL-BiOI demonstrated moderate activity (Figure 5c). These results further highlight the superior ability of the PCL-BT scaffold to generate •OH, which can be attributed to enhanced charge separation and oxygen-vacancy-mediated catalysis.

ESR spectroscopy validated the generation of singlet oxygen (^1^O_2_). Figure 5d presents a diagnostic 1:1:1 triplet signal detected in the PCL-BT scaffold under LIU irradiation using 2,2,6,6-tetramethylpiperidine (TEMP) as the spin trap, demonstrating its capacity to generate singlet oxygen (^1^O_2_) [29]. Singlet oxygen (^1^O_2_) production under LIU was quantitatively monitored using 1,3-diphenylisobenzofuran (DPBF), a selective ^1^O_2_ chemical probe. The absorbance decline of DPBF at 425 nm (Figure 5e) reflects ^1^O_2_ generation via piezocatalytic activity. With prolonged LIU exposure (0–10 min), the PCL-BT scaffold exhibited a marked reduction in DPBF absorbance, whereas negligible changes were observed for PCL and PCL-BiOI groups (Figure 5f). This significant difference highlights the pivotal role of the PCL-BT heterostructure in promoting electron–hole separation and facilitating oxygen capture for subsequent ^1^O_2_ production [30,31]. The enhanced charge separation efficiency, driven by interfacial polarization and oxygen-vacancy-mediated carrier dynamics in BT, facilitates efficient ROS generation. Collectively, these results demonstrate PCL-BT’s efficacy as a high-performance sonosensitizer, exploiting dual-pathway ROS generation (•OH and ^1^O_2_) to combat deep tissue infections while minimizing off-target effects.

### 3.4. In Vitro Antibacterial Properties of the Scaffolds

Bacterial infections during tissue regeneration often lead to delayed healing or severe inflammatory responses. Under LIU, the PCL-BT scaffold efficiently generated multiple reactive oxygen species (ROS) with burst kinetics. This potent ROS generation, confirmed by the sonodynamic assessment, indicates superior antibacterial activity. To verify this hypothesis, the antibacterial efficacy of PCL-BT scaffolds was systematically evaluated against clinically prevalent pathogens: Gram-negative *Escherichia coli* (ATCC 25922) and Gram-positive *Staphylococcus aureus* (ATCC 25923) strains. Bacterial viability assays were conducted under LIU conditions (1.0 W/cm^2^, 10 min), comparing PCL-BT with control groups (PCL and PCL-BiOI) [32].

**Figure 6:**
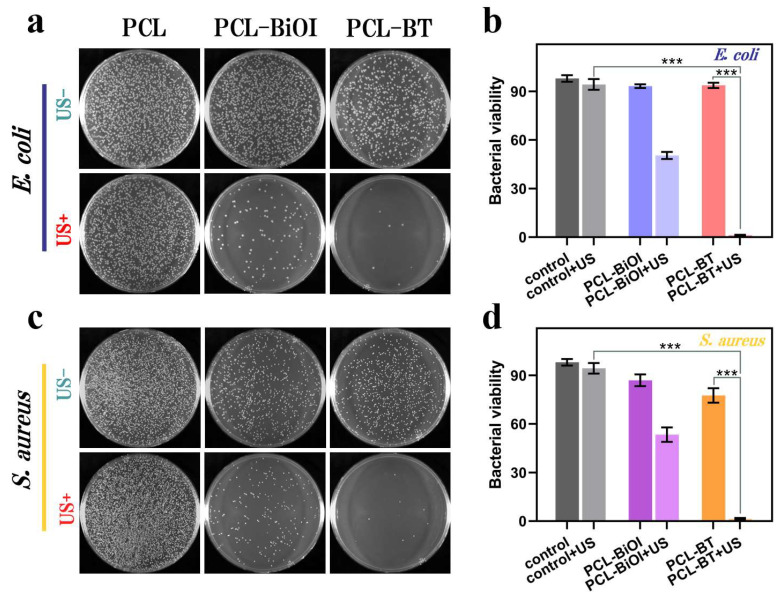
In vitro antibacterial tests: (**a**) Pictures of bacteriological colonies of *E. coli*, and (**b**) antibacterial ratio of *E. coli* (n = 3). (**c**) Corresponding *S. aureus* were treated with different samples, and (**d**) corresponding survival rates of *S. aureus*. (n = 3). Data are presented as mean ± SD (n = 3). *p*-values are assessed by one-way ANOVA analysis; ***: *p* < 0.001.

The antibacterial efficacy of PCL, PCL-BiOI, and PCL-BT scaffolds against *E. coli* and *S. aureus* was assessed via plate counting following 10 min low-intensity ultrasound exposure (1.0 W/cm^2^). As shown in Figure 6a,c, bacterial survival rates were quantified under LIU-treated (US+) and untreated (US−) conditions. For *E. coli*, agar plates without LIU exhibited high bacterial viability across all groups, with abundant colony-forming units (CFUs) observed (Figure 6a). Notably, PCL-BT scaffolds showed no significant antibacterial activity in the absence of LIU. However, under LIU, both PCL-BiOI and PCL-BT scaffolds demonstrated markedly reduced bacterial survival rates. Notably, PCL-BT achieved nearly complete bacterial eradication post LIU, with a 98.87% reduction in colony-forming unit (CFU) counts compared to controls. A similar trend was observed for *S. aureus* (Figure 6c). While untreated groups displayed negligible antibacterial effects, LIU-activated PCL-BT scaffolds exhibited exceptional bactericidal activity, suppressing 98.51% of *S. aureus* colonies. This enhanced efficacy originates from concurrent defect-driven ROS generation (•OH/^1^O_2_) and improved charge carrier separation in PCL-BT during LIU exposure [33,34,35]. The hierarchical design of PCL-BT has achieved an LIU-triggered and ROS-driven antibacterial effect, enabling it to have excellent applications in the treatment of acute bacterial infections.

### 3.5. Extracellular Bacterial ROS Detection

To assess rapid intracellular ROS induction in *E. coli* and *S. aureus* by PCL-BT scaffolds under short-duration LIU, we monitored ROS production using 2’,7’-dichlorodihydrofluorescein diacetate (DCFH-DA) [36,37]. Bacterial esterases deacetylate this probe, forming the non-fluorescent DCFH. Subsequent oxidation by reactive oxygen species (ROS) converts DCFH into the green-fluorescent compound DCF. 

**Figure 7:**
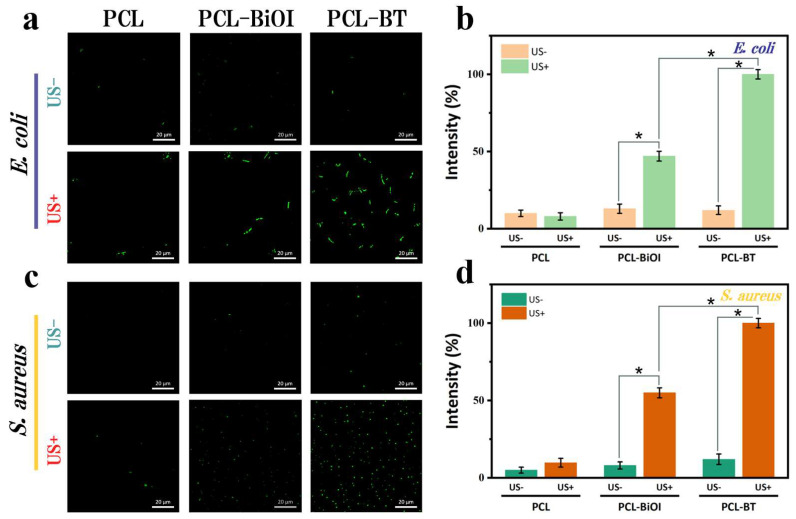
(**a**) Fluorescence microscopy images of *E. coli* stained with DCFH-DA (green fluorescence) for ROS detection, and (**b**) the corresponding fluorescence intensity test results. (**c**) Fluorescence microscopy images of *S. aureus* stained with DCFH-DA for ROS detection, and (**d**) the corresponding fluorescence intensity test results. (where * represents a significant difference at *p* < 0.05; US- and US+ mean in the absence and presence of ultrasound, respectively).

PCL, PCL-BiOI, or PCL-BT scaffolds were individually immersed in the bacterial suspension, incubated for 6 hours, and divided into LIU-treated (US+, 1.0 W/cm^2^, 10 min) and untreated (US−) groups. As shown in Figure 7a,c, negligible green fluorescence was observed in US− groups for both bacteria. Under LIU, PCL scaffolds exhibited minimal fluorescence, while PCL-BiOI showed moderate signals [38,39]. Notably, PCL-BT scaffolds generated the most intense green fluorescence, indicating the highest ROS accumulation. As shown in Figure 7b,d, we further quantified the ROS level by normalizing the DCF intensity. Comparison of fluorescence images and quantitative analysis between US+ and US- groups revealed that PCL-BT generated the highest ROS levels. This enhancement is attributed to improved charge separation efficiency and interfacial charge redistribution, driven by dual electric fields at the BiOI/Ti_3_C_2_ heterojunction. Consequently, the augmented piezoelectric properties of BiOI enable rapid, short-term ROS burst kinetics.

### 3.6. In Vitro Antibacterial Activity Testing

To further investigate the pronounced antibacterial efficacy of PCL-BT scaffolds, we performed live/dead staining assays on *E. coli* and *S. aureus* [40,41]. Bacterial viability was determined through dual-fluorescence staining: 4′,6-diamidino-2-phenylindole (DAPI) labeling total bacteria (blue fluorescence) and propidium iodide (PI) identifying dead bacteria (red fluorescence). 

**Figure 8:**
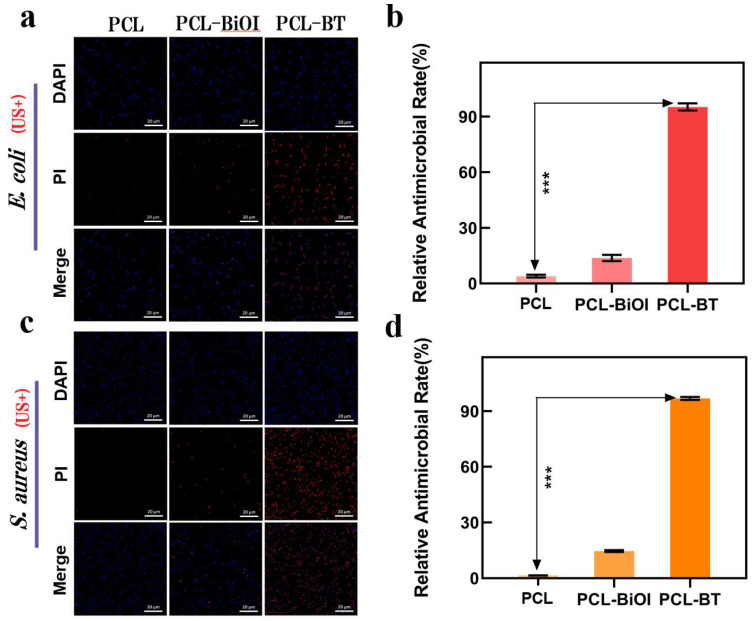
(**a**) LIVE/DEAD staining of *E. coli*. The blue fluorescence: DAPI (representing all bacteria); the red fluorescence: PI (representing dead bacteria). (**b**) The relative antimicrobial rate of *E. coli*. (**c**) Staining of *S. aureus* from different samples. (**d**) The relative antimicrobial rate of *S. aureus*. Data expressed as the mean ± SD (n = 3); *** *p* < 0.001 (Student’s *t*-test).

As shown in Figure 8a,c, ubiquitous blue fluorescence across all groups confirmed substantial bacterial presence. In the PCL control group, negligible red fluorescence under LIU demonstrated ineffective bacterial eradication. PCL-BiOI scaffolds exhibited moderate red fluorescence, indicating partial bactericidal activity. Notably, LIU-treated PCL-BT scaffolds exhibited predominantly red fluorescence, suggesting nearly complete bacterial inactivation. Figure 8b,d quantitatively corroborate these findings through fluorescence intensity measurements. These results, consistent with the colony-counting assays and SEM/TEM analyses, collectively validate the robust antimicrobial properties of LIU-activated PCL-BT scaffolds.

### 3.7. In Vitro Cytocompatibility

**Figure 9:**
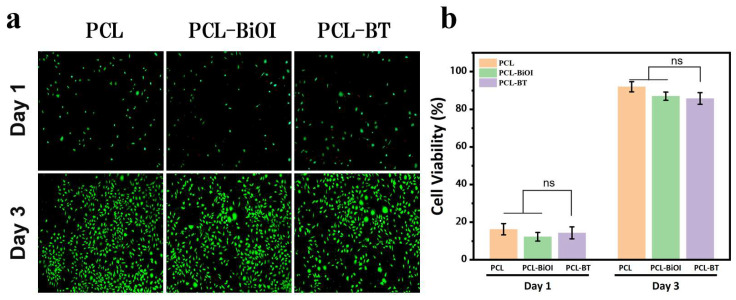
(**a**) Live/dead fluorescence staining images of L929 cells on different scaffolds for 1 day and 3 days (Calcein: live cells, green; PI: dead cells, red), and (**b**) corresponding quantitative results of cell survival rates for L929 cells stained live/dead for 1 day and 3 days (n = 3 for each group; *p*-value was evaluated using one-way analysis of variance; ns = no significant difference).

The above experiments verified the excellent antibacterial performance of the scaffold materials. As a bioengineering implant scaffold, good biocompatibility is essential. Therefore, we evaluated the effect of different scaffolds on L929 fibroblast proliferation using live/dead cell staining. The experiment used a live/dead cell stain to detect the cell viability of L929 cells after 1 day and 3 days of cultivation on each scaffold material. Through fluorescence microscopy, it was observed that cells on all scaffolds exhibited strong green fluorescence (indicating live cells) and healthy morphology. Few dead cells (red fluorescence) were observed (Figure 9a). We conducted corresponding quantitative analysis of the live/dead staining of L929 cells, and the results are shown in Figure 9b. Over time, cell numbers increased on all scaffolds. High cell viability was maintained on both day 1 and day 3.

## 4. Conclusions

In this work, we fabricated an ultrasound-responsive BiOI/Ti_3_C_2_ heterojunction via in situ growth on MXene nanosheets and integrated it into 3D-printed PCL scaffolds. The heterojunction creates a unidirectional built-in electric field that drives piezoelectric electrons from BiOI to the conductive Ti_3_C_2_ matrix, while interfacial oxygen vacancies act as electron traps. This synergistic mechanism significantly suppressed charge recombination, leading to a 2.8-fold increase in piezoelectric current density under low-intensity ultrasound (LIU), indicating enhanced charge separation. Consequently, extremely high yields of reactive oxygen species (ROS) were generated within 10 min, endowing the scaffold with exceptional antibacterial efficacy: 98.87% eradication of *E. coli* and 98.51% of *S. aureus*. The scaffold also exhibited excellent cytocompatibility with L929 fibroblasts. The designed PCL-BT scaffold, combining efficient piezocatalytic antibacterial action with inherent biocompatibility, represents a promising strategy for treating deep tissue implant infections and promoting bone regeneration. In future research, we will conduct in vivo studies to facilitate future clinical applications.

## Data Availability

The original contributions presented in this study are included in the article. Further inquiries can be directed to the corresponding authors.
